# Intraindividual Fluctuation in Optimism Under Daily Life Circumstances: A Longitudinal Study

**DOI:** 10.1007/s42761-023-00224-y

**Published:** 2023-11-20

**Authors:** Kanji Shimomura, Kenji Morita, Yuki Nishiguchi, Jeff C. Huffman, Rachel A. Millstein

**Affiliations:** 1 Physical and Health Education, Graduate School of Education, The University of Tokyo, Tokyo, Japan; 2 International Research Center for Neurointelligence (WPI-IRCN), The University of Tokyo, Tokyo, Japan; 3 Faculty of Education, Chiba University, Chiba, Japan; 4 Department of Psychiatry, Massachusetts General Hospital, Boston, MA USA; 5 Harvard Medical School, Boston, MA USA

**Keywords:** State optimism, Trait optimism, Intraindividual fluctuation, Longitudinal survey, Mood

## Abstract

Optimism is typically conceptualized as a relatively static tendency regarding positive expectations about one’s future. However, recent studies suggest that optimism may meaningfully fluctuate within individuals over time. To date, little is known about the characteristics of such state optimism and potential cultural difference in state optimism. Accordingly, we developed a Japanese version of the State Optimism Measure (J-SOM) and examined its validity and the nature of intraindividual state optimism fluctuations; we also examined relationships between the J-SOM and other measures of mental health, including trait optimism. We conducted two online longitudinal surveys with different time intervals (weekly, *n* = 97; monthly, *n* = 99) targeting university students. Results were largely consistent between the two surveys. We confirmed high factor validity and internal consistency of the J-SOM. The J-SOM showed significant correlations in expected directions with other measures such as depressive mood and subjective happiness. In addition, intraindividual changes in the J-SOM were associated with changes in mood and quality of daily life. Importantly, these associations between intraindividual change in optimism and in other variables were minimal for trait optimism. We also found that state optimism, compared with trait optimism, tended to show larger intraindividual changes over 1, 2, 3, 4, and 8 weeks. In summary, this study developed a translated version of the SOM and validated it, and then showed, for the first time, that state optimism can fluctuate within individuals in daily life over a span of several weeks.

**Supplementary Information:**

The online version contains supplementary material available at 10.1007/s42761-023-00224-y.

Optimism is typically defined as a tendency to have general positive expectancy about one’s future (Carver et al., 2010; Carver & Scheier, 2014). High dispositional optimism predicts future lower depressive tendency (Vickers & Vogeltanz, 2000), lower mortality rates caused by stroke (Kim et al., 2011), and other cardiovascular diseases (Tindle et al., 2009). These and many other studies examining the relationship between optimism and health or well-being indicate that optimism is an important factor for both mental and physical health (Carver & Scheier, 2014; Rius-Ottenheim et al., 2012).

As indicated by the term “dispositional,” most previous studies have treated an optimistic tendency as a stable factor that does not vary over time within the same person. However, it is suggested that optimism as a generalized expectancy can fluctuate within individuals depending on time, external circumstances, and one’s experiences (Luthans & Youssef, 2007). In fact, several studies have revealed that dispositional optimism measured over long periods can show relatively low test-retest correlation. For example, in Lucas et al. (1996), the 2-year test-retest correlation of the Life Orientation Test (Scheier & Carver, 1985) was 0.58. Likewise, Segerstrom (2007) reported that the 10-year test-retest reliability of trait optimism was only 0.35. Furthermore, optimism may be modifiable. A meta-analysis (Malouff & Schutte, 2017) found that psychological interventions could increase optimism measured by Life Orientation Test-Revised (Scheier et al., 1994). Some such studies included in the meta-analysis were successful in increasing optimism after as few as 1 to 2 weeks of psychological intervention (Meevissen et al., 2011; Peters et al., 2013). These empirical findings indicate that optimism, even measured as a trait, can fluctuate within individuals over time or circumstance, and that optimism might have more state-like aspect than previously thought.

Based on this idea, a scale specifically designed to measure state optimism was developed (Millstein et al., 2019). This State Optimism Measure (SOM) aims to quantify the potential within-individual dynamics of optimism as the context-independent, generalized positive expectancies for the future. To emphasize the state-based nature of the scale, items include a stem instructing respondents to consider their answers “right now, at the present moment.” The validity of the SOM is supported not only by cross-sectional data (Millstein et al., 2019), but also by a longitudinal survey (Hoeppner et al., 2022). Specifically, the SOM showed greater decline from pre- to post-COVID-19 pandemic in a secondary analysis compared with the LOT-R (Hoeppner et al., 2022). This result highlights the capability of the SOM to capture longitudinal change in optimism and the fact that optimism can change within individuals.

Despite this prior work, several issues remain to be solved related to state optimism. One is the need for more culturally diverse findings. The participants in the previous surveys (Hoeppner et al., 2022; Millstein et al., 2019) were limited to residents in the USA. Given that the level of trait optimism and its relation to other variables differ depending on the population or culture (Chang, 1996; Wang et al., 2022; You et al., 2009), it is conceivable that the characteristics of state optimism also differ between different cultures.

The second is the need for further investigation to elucidate the longitudinal nature of state optimism. Although there is one longitudinal study using the SOM (Hoeppner et al., 2022), it focused on the change in state optimism between two time points under the special condition (i.e., COVID-19) and in a specific population (smokers in an online positive psychology intervention). Therefore, it is still not clear whether and how state optimism fluctuates within those under daily life circumstances. Given the large number of studies showing an association between optimism and mental/physical health, not only the level of state optimism at a specific time point, but also its within-individual change might also have a unique effect on a wide range of physical and mental diseases. This idea is bolstered by prior research showing that future reward expectancy is closely related to momentary happiness and mood (Bennett et al., 2022; Rutledge et al., 2014) and that positivity of future outcome expectancy is an important component of hopelessness (Abramson et al., 1989; Alloy et al., 1988). Clarifying the nature of intraindividual fluctuation in state optimism can, therefore, facilitate mechanistic understanding of depression and associated negative affective states.

Therefore, the aim of the current study was twofold. The first one was to develop and validate the Japanese version of the SOM, which is expected to promote future Japanese study on state optimism. The second was to clarify the longitudinal characteristics of state optimism. More specifically, we aimed to examine how large and over what time span state optimism fluctuates within individuals in daily life. To achieve these aims, we developed the Japanese SOM and conducted two longitudinal surveys, which differed in their time intervals, targeting university students in Japan.

## Method

### Translation

Referring to the guideline of the International Society for Pharmacoeconomics and Outcomes Research (ISPOR) task force (Wild et al., 2005), the translation procedure of the SOM was conducted as follows: First, the first author (KS) translated the original SOM into Japanese in consultation with other two Japanese authors (KM and YN). Next, it was backtranslated by a commercial bilingual team who did not know the content of the original scale. Then, the authors of the original scale (JCH and RAM) compared it with the original scale and checked the accuracy of the descriptions and meaning of each item. Based on the original scale authors’ comments, the Japanese authors revised the descriptions. Finally, four Japanese graduate students majoring in psychology, who were unrelated to this research, answered the questionnaire and confirmed that there were no problems with the relevance and clarity of the items. All items of the finalized version of the Japanese SOM can be seen at https://osf.io/rp5vh/.

### Participants

The current study consisted of two online longitudinal surveys, which differed in the number and the interval of their distribution waves. The first one was a 4-wave 1-week interval survey (hereafter “1w survey”) and the second one was a 3-wave 1-month interval survey (hereafter “1m survey”). In both surveys, undergraduate and graduate students were recruited via a website (https://www.jikken-baito.com) that provides information on participation in psychological experiments and surveys in Japan. Those who participated in one survey were not allowed to participate in the other. The rationale for selecting undergraduate and graduate students as participants was that they are generally in the period in their lives when they have to think seriously and concretely about their future (i.e., how they should live their lives), and thus, the positivity/negativity of their general predictions about the future (i.e., optimism) was expected to tend to fluctuate in their daily lives. Also, university presents many new challenges and situations (e.g., exams, social support), which we thought would influence state optimism.

Due to limited prior research in these areas, it was difficult to determine the expected effect sizes for the association between intraindividual change in state/trait optimism and in other variables prior to the surveys. We therefore aimed to have a sample size that would allow us to detect medium correlations between variables. Based on a guideline of Cohen (Cohen, 1988), we interpreted *r* = .3 as a medium effect size. Power analysis using pwr package of R showed that with a correlation of .3 and alpha of .95, 84 participants were required to achieve power of .8. Taking this result and possible dropouts (about 10%) into consideration, we planned to recruit at least 100 participants in both surveys.

### Procedure

Both longitudinal surveys were conducted online using Google Forms. In the 1w survey, participants selected 1 day of week on which they would like to respond to the questionnaire prior to the start of the survey and responded to the form on that day for 4 consecutive weeks. In the 1m survey, participants were asked to respond to the form for 3 consecutive months during the period (3 days) which we specified at each month. The 1w survey was conducted from June to July 2022, and the 1m survey was conducted from July to September 2022.

### Measures

#### State Optimism

State optimism was assessed with a Japanese version of the State Optimism Measure (J-SOM). The J-SOM is a five-point scale consisting of seven items (e.g., “I am expecting things to turn out well”) that assess the current degree of general positive expectancy for future. Note that none of the items ask about expectations regarding specific events or situations. Participants were instructed to answer each item based on their feeling in the “present moment.”

#### Trait Optimism

Trait optimism was assessed with a Japanese version of the Life Orientation Test-Revised (Sakamoto & Tanaka, 2002; Scheier et al., 1994). The LOT-R is a five-point scale consisting of ten items, including three items measuring optimism (e.g., “In uncertain times, I usually expect the best”), another three items measuring pessimism (e.g., “I hardly ever expect things to go my way”), and four filler items (e.g., “It’s easy for me to relax”). We reverse coded the three pessimism items and treated the sum of them and three optimism items as a total trait optimism score. Participants were instructed to answer each item based on how they “usually” feel.

#### Global Subjective Happiness

Global subjective happiness was assessed with a Japanese version of the Subjective Happiness Scale (Lyubomirsky & Lepper, 1999; Shimai et al., 2004). The SHS is a seven-point scale consisting of four items (e.g., “Some people are generally very happy. They enjoy life regardless of what is going on, getting the most out of everything. To what extent does this characterization describe you?”).

#### Depressive, Positive, and Anxiety Mood

Depressive, positive, and anxiety mood were assessed with the Depression and Anxiety Mood Scale (DAMS; Fukui, 1997), which is a Japanese scale. The DAMS is a seven-point scale consisting of nine items, including three items each for measuring depressive (e.g., “Depressed”), positive (e.g., “Happy”), and anxiety mood (e.g., “Nervous”). Participants were asked to answer the extent to which each statement applied to their mood in the last 2 to 3 days.

#### Depressive Symptoms

Depressive symptoms in the last week were assessed with the Japanese version of the Center for Epidemiological Studies Depression Scale (CES-D; Radloff, 1977; Shima et al., 1985). Participants answered how often they had experienced the 20 depressive symptoms (e.g., “I felt lonely”) in the last week using a four-point scale.

#### Quality of the Previous Week/Month

In order to assess the total general influence of everyday experience on optimism, we asked participants to answer the subjective quality of the last week/month in the 1w/1m survey, respectively. Specifically, participants answered to one item “how good/bad their last week/month was compared to their usual life” on a scale of 1 to 9.

### Analysis

Since the surveys were conducted completely online, we included one “trick question” (or a “confirmation question”) in questionnaires in all waves to assess if participants concentrated on answering questions. Specifically, the wording was as follows: “This item is for confirmation. Please do not answer anything to this item.” In both surveys, participants who answered anything to this question at any wave were excluded from the analysis (four and two participants were excluded in 1w and 1m survey, respectively). We adopted a *p*-value of 0.05 as a significance level of all statistical tests in this study.

#### Factor Validity and Internal Consistency

For factor validity of the J-SOM, we conducted confirmatory factor analysis to see the fit of the one-factor model assumed in the original SOM. To see the fit of the model, we applied the criteria suggested in Hu and Bentler (1999): Comparative Fit Index (CFI) > 0.90 (acceptable), Tucker–Lewis Index (TLI) > 0.90 (acceptable), root mean square error of approximation (RMSEA) < 0.06, and standardized root mean square residual (SRMR) < 0.08. We planned to conduct exploratory factor analysis only if the one-factor model showed unacceptable fit to the data. Internal consistency of the items that were confirmed to fit the one-factor model was assessed using Cronbach’s $\alpha .$ In this study, $\alpha \ge .85$ was considered excellent consistency, according to the categorization of Haertel (2006).

#### Construct Validity

To assess the construct validity of the J-SOM, we applied two criteria: (1) if J-SOM shows positive correlation with conceptually similar measures and shows negative correlation with conceptually opposite measures, and (2) if the intraindividual change in J-SOM, compared with the change in LOT-R, correlates more strongly with the intraindividual change in mood, in depressive symptoms, and in the quality of the previous week/month.

As for criterion (1), we calculated the zero-order correlation coefficients between J-SOM and other variables at wave 1 of 1w and 1m survey. For criterion (2), we examined the association between intraindividual change in 1 week and 1 month of J-SOM/LOT-R and of other variables. 1w survey and 1m survey had four and three waves each; thus, we obtained three “1-week change scores” and two “1-month change scores.” To integrate association of these values at multiple time points, we used a linear mixed model assuming the random effect of time. Specifically, we regressed standardized (i.e., z-score of) change in trait/state optimism scores by standardized change in other scores, with assumption of random effect of time on regression coefficients. A fixed effect (i.e., standardized regression coefficient) of independent variables on trait/state optimism was interpreted as the strength of the association between change in optimism and in other variables.

#### Examination of the Longitudinal Nature of State Optimism

To see whether state optimism changes within individual, we examined the amount of intraindividual absolute change in J-SOM at each interval (1, 2, and 3 weeks for 1w survey, and 4 and 8 weeks for 1m survey). In this part, when more than one data set of absolute changes at a given interval was available, we only showed the data from the earliest wave in the main text for simplicity. In other words, for 1w survey, we treated “wave 2 — wave 1” score and “wave 3 — wave 1” score as “1-week change score” and “2-week change score,” respectively. Similarly, for 1m survey, we treated “wave 2 — wave 1” score as “1-month (4-week) change score.” Because the assumption of normality of the difference in absolute change in mean score of LOT-R and J-SOM was rejected at all intervals according to the results of the Shapiro-Wilk test, we used non-parametric tests for all tests of the absolute change. We first conducted one sample Wilcoxon’s signed-rank test to see whether state optimism (J-SOM) showed statistically significant intraindividual change in each interval of the two surveys. In addition, to examine whether and on what time span state optimism shows greater intraindividual change compared with trait optimism, we tested whether absolute change in J-SOM was larger than that in LOT-R at each interval using the paired Wilcoxon’s signed-rank test. All *p*-values for Wilcoxon’s tests were adjusted with the Holm method.

#### Analysis with Missing Values

Because about 15% of the respondents dropped out in 1w survey, we adopted a multiple imputation method in the analysis with the linear mixed model and in the non-parametric tests when needed (i.e., when there were missing values in the variables used in the analysis). As for the linear mixed model, we reported the pooled results of 100 imputed datasets. As for the Wilcoxon’s tests, because (to the best of our knowledge) there is no validated way to integrate the results of non-parametric tests of imputed datasets, we first conducted the analysis with the original dataset using listwise deletion method. Then, we repeated the same Wilcoxon’s tests with 1000 different imputed datasets and calculated the *Z*-value of each test. The average *Z*-value of 1000 tests was used as information that supports the validity of the original tests. For 1m survey, on the other hand, we omitted the data of dropouts from the analysis because the proportion of dropouts was small (2.1%). All data analysis was conducted with R version 4.0.3, and all data and the codes for analysis or drawing figures are available at https://osf.io/rp5vh/.

## Results

### Characteristics of Participants

In total, 101 students participated at wave 1 of both 1w survey and 1m survey. Of 101 students, four and two participants in 1w and 1m survey, respectively, were excluded from the analysis since they were thought to respond inappropriately in at least one of the waves (see “Analysis” for the specific criteria for exclusion). Table 1 shows the characteristics of the participants included in the data analysis. In 1w survey, mean age was 21.87 (*SD* = 2.88). Of 97 participants, 38 were female and 59 were male. Ninety-four, 91, and 86 students responded at waves 2, 3, and 4, respectively, and 83 (85.6%) students responded in all four waves. In 1m survey, mean age was 21.28 (*SD* = 2.50). Of 99 participants, 49 were female, 48 were male, 1 was none of both, and 1 selected no response. Ninety-seven and 95 students responded at waves 2 and 3, respectively, and 95 (97.9%) students responded to all three waves.

**Table 1:** Characteristics of participants who were included in the analysis

Sample characteristics	Survey 1 (1w survey)	Survey 2 (1m survey)
Number of participants at wave 1	97	99
Number of participants at wave 2	94	97
Number of participants at wave 3	91	95
Number of participants at wave 4	86	No wave
Mean age at wave 1 (SD)	21.87 (2.88)	21.28 (2.50)
Gender at wave 1	F: 38, M: 59	F: 49, M: 48, O: 1, N: 1
Number of people who participated in all waves (%)	83 (85.6%)	95 (97.9%)

*F* female, *M* male, *O* other, *N* No answer

### Confirmatory factor analysis

We conducted confirmatory factor analysis to see the fit of the one-factor model assumed in the original SOM to the data of the wave 1 of 1w and 1m survey. The one-factor model showed excellent fit in both surveys (1w survey: ${\chi }^{2}\left(9\right)=$ 8.218, *p* = .512, CFI = 1.000, TLI = 1.004, RMSEA = 0.000, SRMR = 0.024, 1m survey: ${\chi }^{2}\left(9\right)=$ 9.267, *p* = .413, CFI = 0.999, TLI = 0.998, RMSEA = 0.017, SRMR = 0.032). Therefore, we did not conduct further exploratory factor analysis.

### Internal Consistency

J-SOM showed high internal consistency in both surveys, indicated by Cronbach’s $\alpha$ of the total score showing .91 for 1w survey and .88 for 1m survey.

### Construct Validity

#### Correlation Between State Optimism and Other Variables

To examine the first criterion, i.e., (1) if J-SOM shows positive correlation with conceptually similar measures and shows negative correlation with conceptually opposite measures, we calculated correlation coefficients between J-SOM and other variables. Table 2 and S1 show a correlation matrix of wave 1 of 1w survey and 1m survey, respectively, with descriptive statistics and alpha coefficients of each scale. As shown in the tables, J-SOM showed significant positive correlation with LOT-R (1w survey: *r* = .68, *p* < .001, 1m survey: *r* = .72, *p* < .001), SHS (1w survey: *r* = .56, *p* < .001, 1m survey: *r* = .49, *p* < .001), positive mood (1w survey: *r* = .56, *p* < .001, 1m survey: *r* = .52, *p* < .001), and the quality of the previous week (1w survey: *r* = .56, *p* < .001, 1m survey: *r* = .56, *p* < .001) and significant negative correlation with depressive mood (1w survey: *r* = − .45, *p* < .001, 1m survey: *r* = − .43, *p* < .001), anxiety mood (1w survey: *r* = − .28, *p* < .05, 1m survey: *r* = − .41, *p* < .001), and CES-D (1w survey: *r* = − .38, *p* < .001, 1m survey: *r* = − .47, *p* < .001), in both surveys.

**Table 2:** Descriptive statistics of each scale and correlation matrix at wave 1 of 1w survey. *J-SOM* Japanese version of State Optimism Measure, *LOT-R* Life Orientation Test-Revised, *SHS* Subjective Happiness Scale, *DAMS-P* Positive Mood subscale of DAMS, *QW* The Quality of the previous Week, *DAMS-D* Depressive Mood subscale of DAMS, *DAMS-A* Anxiety Mood subscale of DAMS, *CES-D* the Center for Epidemiological Studies Depression Scale

	Mean (*SD*)	Range	α	J-SOM	LOT-R	SHS	DAMS-P	QW	DAMS-D	DAMS-A	CES-D
State optimism (J-SOM)	20.13 (6.82)	7–35	.91	-	.65^***^	.56^***^	.56^***^	.56^***^	− .45^***^	− .28^**^	− .42^***^
Trait optimism (LOT-R)	18.34 (4.35)	6–30	.71		-	.52^***^	.48^***^	.42^***^	− .37^**^	− .21^*^	− .35^***^
Subjective happiness (SHS)	17.59 (2.90)	4–28	.85			-	.47^***^	.45^***^	− .22^*^	.04	− .32^**^
Positive mood (DAMS-P)	12.80 (4.15)	3–21	.87				-	.77^***^	− .61^***^	− .34^**^	− .51^***^
Quality of the previous week (QW)	5.36 (1.87)	1–9	-					-	− .59^***^	− .29^**^	− .50^***^
Depressive mood (DAMS-D)	11.32 (4.86)	3–21	.87						-	.63^***^	.67^***^
Anxiety mood (DAMS-A)	14.13 (4.32)	3–21	.85							-	.55^***^
Depressive symptoms (CES-D)	16.67 (9.97)	0–60	.88								-

*p < .05; **p < .01; ***p < .001

#### Association Between Intraindividual Change in Optimism and in Other Variables

**Fig. 1:**
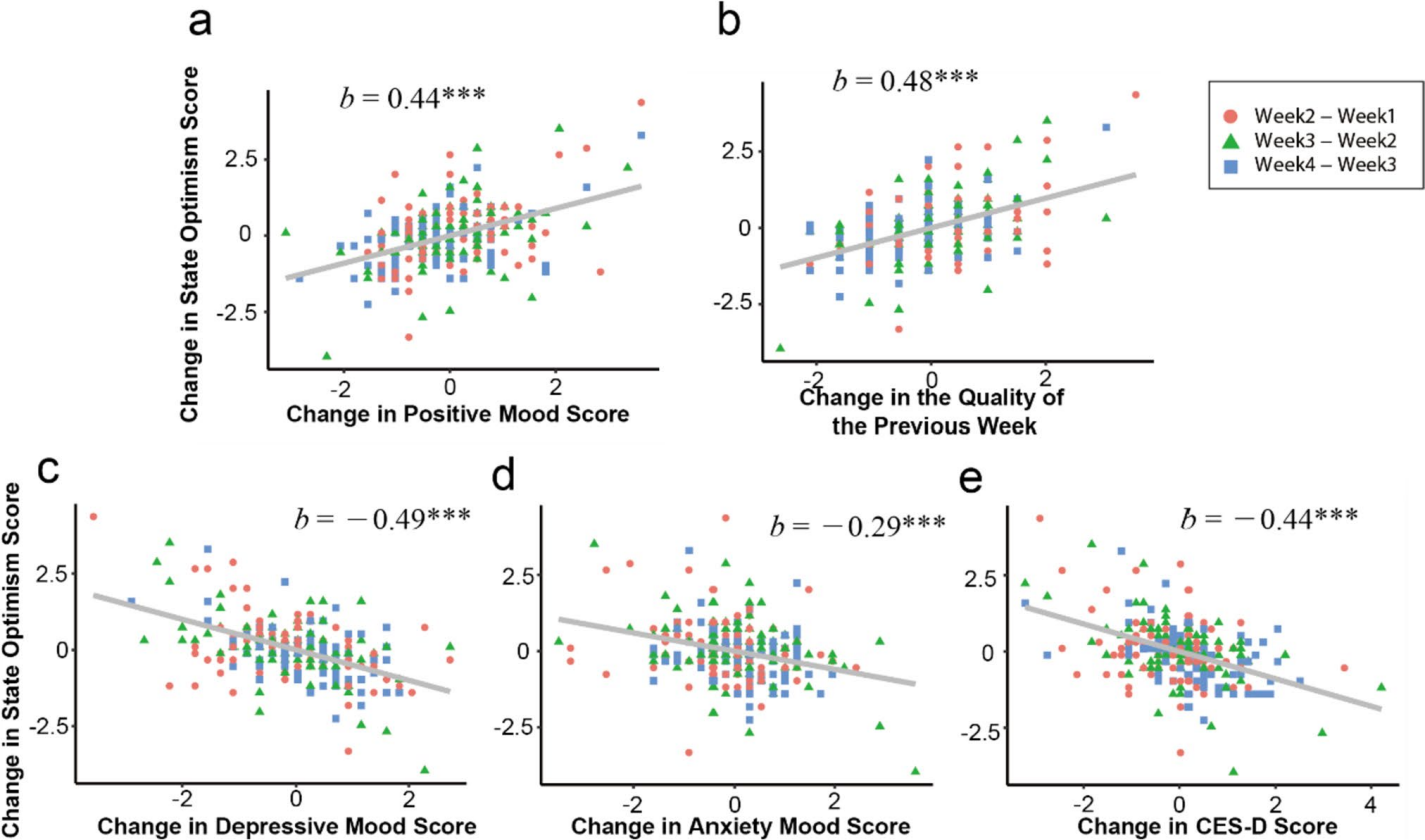
Association between the intraindividual change in 1 week in J-SOM (state optimism) and in positive mood (**a**), in the quality of the previous week (**b**), in depressive mood (**c**), in anxiety mood (**d**), and in depressive symptoms (**e**). Magenta circles, green triangles, and blue rectangles represent the change in scores between “Wave 1 and Wave 2,” “Wave 3 and Wave 2,” and “Wave 4 and Wave3”, respectively (the same applies to Fig. 2). Asterisks and daggers represent the *p*-values of fixed effect of each independent variables (the same applies to Fig. 2, S1, and S2; *p < .05; **p < .01; ***p < .001)

**Fig. 2:**
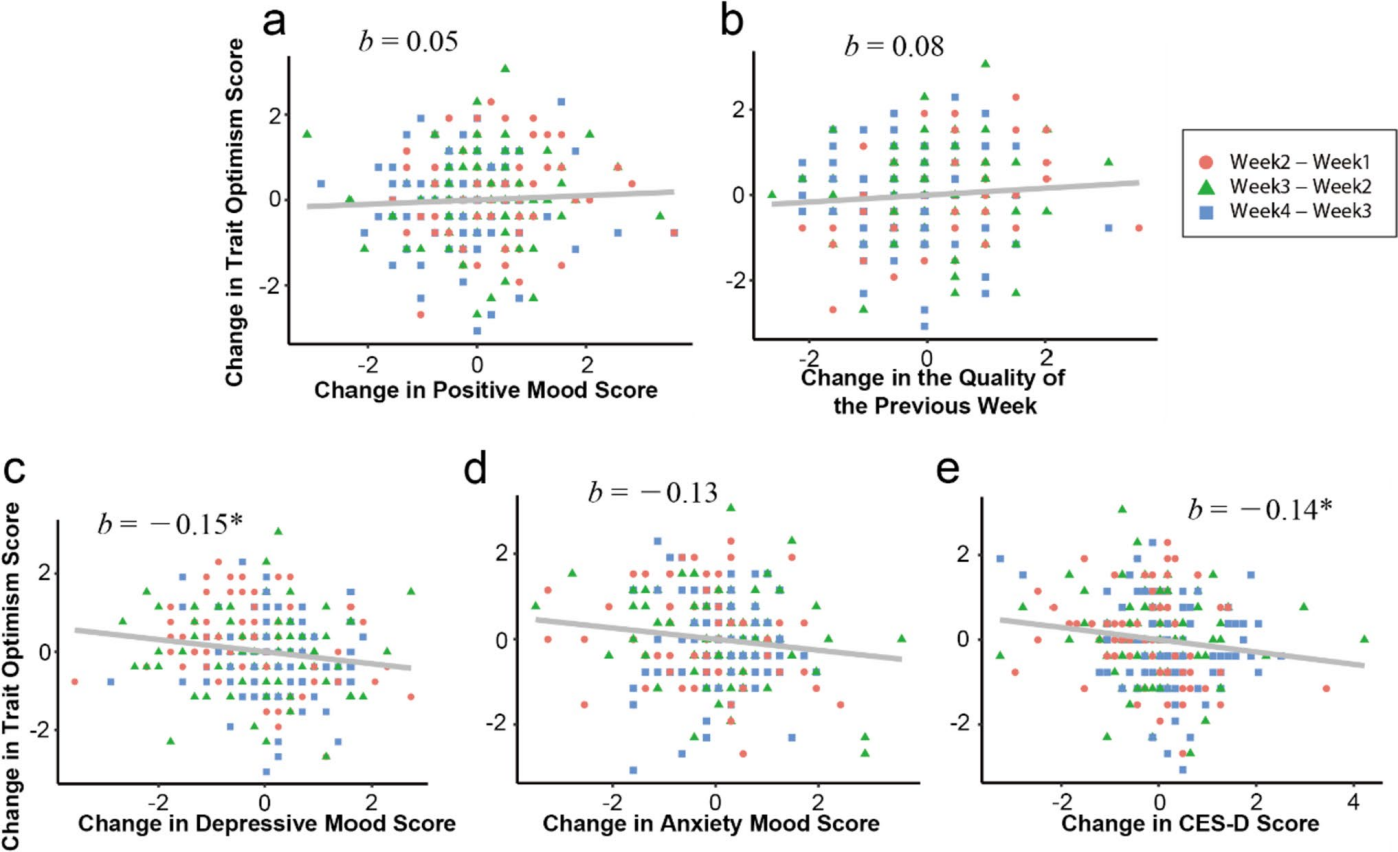
Association between the intraindividual change in 1 week in LOT-R (trait optimism) and in positive mood (**a**), in the quality of the previous week (**b**), in depressive mood (**c**), in anxiety mood (**d**), and in depressive symptoms (**e**)

If J-SOM captures state optimism that fluctuates within the individual, its *intraindividual change* is expected to correlate with the change in other variables that represent the internal state of the individual and with the change in the quality of daily life. In addition, such association is expected to be smaller for LOT-R, since it is assumed to measure the time-invariant trait of the individual. We examined these using a linear mixed model with the random effect of time (see “Method” for the detail). For 1w survey, 1-week change of J-SOM showed significant positive association with the change in positive mood (b* = .44, *p* < .001, 95%CI [.33, .55]) and the quality of the previous week (b* = .48, *p* < .001, 95%CI [.37, .60]), and significant negative association with the change in depressive mood (b* = − .49, *p* < .001, 95%CI [− .60, − .39]), anxiety mood (b* = − .29, *p* < .001, 95%CI [− .43, − .14]), and depressive symptoms (b* = − .44, *p* < .001, 95%CI [− .56, − .32], Fig. 1). One-week change in LOT-R, on the other hand, showed significant but weak association only with change in depressive mood (b* = − .15, *p* = .03, 95%CI [− .28, − .02]) and depressive symptoms (b* = − .14, *p* = .03, 95%CI [− .27, − .01]) and did not show significant association with change in other variables (positive mood: b* = .05, *p* = .44, 95%CI [− .07, .17]; quality of the previous week: b* = .08, *p* = .28, 95%CI [− .06, .22]; anxiety mood: b* = − .13, *p* = .05, 95%CI [− .26, .00], Fig. 2).

These results were similar for the 1m survey, although the associations between change in J-SOM and in other variables were slightly weaker than those observed in 1w survey. Specifically, 1-month change of J-SOM showed weaker but significant positive association with the change in positive mood (b* = .31, *p*<.01, 95%CI [.14, .48]) and the quality of the previous month (b* = .31, *p*<.01, 95%CI [.14, .47]) and weaker but significant negative association with the change in depressive mood (b* = − .20, *p*<.01, 95%CI [− .34, − .06]) and anxiety mood (b* = − .23 *p*<.01, 95%CI [− .37, − .10]), while the association with the change in depressive symptoms did not reach significance (b* = − .32, *p* = .08, 95%CI [− .77, .12], Fig. S1). On the other hand, 1-month change in LOT-R did not show significant association with change in any of the other variables (positive mood: b* = .09, *p* = .20, 95%CI [− .05, .24]; quality of the previous month: b* = .18, *p* = .06, 95%CI [− .02, .38]; depressive mood: b* = − .16, *p* = .36, 95%CI [− 1.16, .85]; anxiety mood: b* = − .14, *p* = .56, 95%CI [− 4.21, 3.93]; depressive symptoms: b* = − .18, *p* = .23, 95%CI [− .78, .41], Fig. S2). Raw correlations between change in optimism and change in other variables are shown in Table S2. In summary, most of the changes in mood, depressive symptoms, and the quality of the previous week/month were associated only with change in J-SOM and not with change in LOT-R.

### Examination of the Longitudinal Nature of State Optimism

**Fig. 3:**
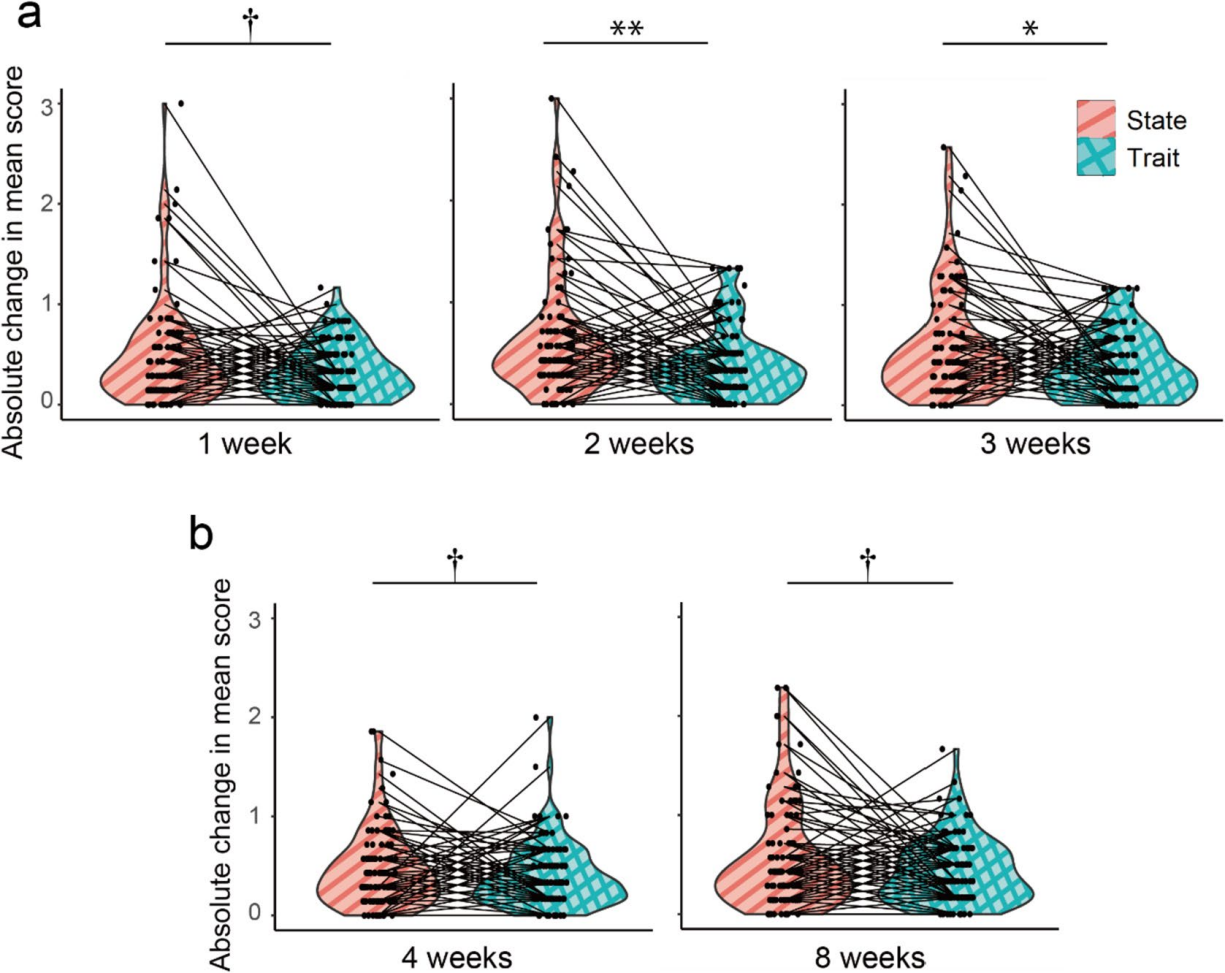
The intraindividual absolute change in J-SOM (state optimism) and LOT-R (trait optimism) in various intervals. Magenta color with stripe patterns represents the score of J-SOM, and cyan color with crosshatch patterns represents the score of LOT-R. **a** Absolute changes in 1w survey. **b** Absolute changes in 1m survey. Asterisks on the horizontal line represent the *p*-values of paired Wilcoxon’s test (†*p* < .10; *p < .05; **p < .01)

In order to examine how large and on what time span state optimism fluctuates within individuals under daily life, we conducted one-sample Wilcoxon’s signed-rank test, using the absolute intraindividual change in J-SOM in various intervals as a dependent variable. The absolute changes in J-SOM were significantly different from zero in all intervals (1 week: *Z* = 7.79, *p*<.001, *r* = .80, 2 weeks: *Z* = 7.78, *p*<.001, *r* = .80 (avg. *Z* = 8.02), 3 weeks: *Z* = 7.64, *p*<.001, *r* = .78 (avg. *Z* = 8.09), 4 weeks: *Z* = 8.02, *p*<.001, *r* = .81, 8 weeks: *Z* = 7.98, *p*<.001, *r* = .80). Note that “avg. *Z*” refers to a mean *Z*-value of 1000 tests with different imputed datasets for 1w survey, and that “*r*” in this section is not a correlation coefficient but an effect size for Wilcoxon’s tests, which is calculated by dividing *Z* by the square root of the sample size *n*. This indicates that state optimism fluctuated within individual in all intervals, but the same test found that the absolute change in LOT-R also differed significantly from zero in all intervals (1 week: *Z* = 7.50, *p*<.001, *r* = .77, 2 weeks: *Z* = 7.50, *p*<.001, *r* = .76 (avg. *Z* = 7.75), 3 weeks: *Z* = 7.14, *p*<.001, *r* = 0.72 (avg. *Z* = 7.60), 4 weeks: *Z* = 7.99, *p*<.001, *r* = .80, 8 weeks: *Z* = 7.88, *p*<.001, *r* = .80). Next, to examine whether such fluctuation in state optimism was larger than that in trait optimism, we compared the degree of the absolute intraindividual change in J-SOM and LOT-R using paired Wilcoxon’s signed-rank test. For 1w survey, the absolute intraindividual changes of J-SOM in 2 weeks (*Z* = 3.16, *p*<.001, *r* = .32, avg. *Z* = 3.28) and 3 weeks (*Z* = 2.39, *p*<.001, *r* = .25, avg. *Z* = 2.58) were significantly larger than those of LOT-R, and there was a trend level difference in changes in 1 week (*Z* = 1.45, *p* = .07, *r* = .15, Fig. 3a). For 1m survey, there was a marginally significant difference in the change in both 4 weeks (*Z* = 1.64, *p* = .10, *r* = .16) and 8 weeks (*Z* = 1.33, *p* = .09, *r* = .13) (Fig. 3b). All median and mean scores of these intraindividual absolute changes in trait/state optimism are shown in table S3. In summary, state optimism showed significant change in all the intervals in this study (i.e., 1, 2, 3, 4, and 8 weeks) within individuals, and such fluctuations were found to be larger than those in trait optimism (LOT-R) at least in trend-level (*p* = .10) in all intervals, with significantly greater changes observed at 2 and 3 weeks.

## Discussion

In this study, we developed and examined the validity of the Japanese version of the State Optimism Measure (J-SOM) and investigated the characteristic of intraindividual fluctuation in state optimism. In summary, the results of our longitudinal surveys supported the validity and single-factor structure of the J-SOM and revealed that state optimism showed change within individuals in the intervals of 1, 2, 3, 4, and 8 weeks. This is the first study to reveal the nature of intraindividual fluctuation in state optimism under daily life without external event that impacts the whole population.

As a result of the confirmatory factor analysis, the one-factor structure of the J-SOM, of the original SOM (Millstein et al., 2019), was supported. At the same time, the J-SOM showed high internal consistency, indicated by high values of Cronbach’s alpha. These results highlight the sufficient factorial validity and reliability of the items of J-SOM. As for the construct validity, the J-SOM showed significant correlations in the expected direction with conceptually similar and opposite measures. In addition, the intraindividual change in the J-SOM showed significant association with the intraindividual changes in mood and the quality of the daily life. Importantly, these associations were minimal for the intraindividual change in LOT-R. This indicates that the J-SOM can capture state-like change in optimism within individuals, which was not captured by LOT-R.

The intraindividual change in J-SOM showed strong association with the change in mood and the quality of the daily life. This result is consistent with the previous suggestions or findings that optimism fluctuates depending on time, situation, and life events (Hoeppner et al., 2022; Luthans & Youssef, 2007; Schwaba et al., 2019). But our result that change in LOT-R did not show significant association with the change in mood in most cases is different from the result observed in Hoeppner et al. (2022), in which change in LOT-R showed moderate correlation with change in positive/negative mood and perceived stress. This discrepancy might be due to the difference in the length of the interval at which the changes were examined. The correlations reported in Hoeppner et al. (2022) were those of the changes in the intervals of 3 months on average, whereas the associations reported in the present study were those of the changes in the intervals of at most 1 month. Although speculative at this stage, if one’s level of trait optimism reflects the long-term average level of state optimism, then it is possible that the correlation between changes in state and trait optimism increases as the interval of the change gets longer, and thus, correlations between change in trait optimism and in other variables approach those between the change in state optimism and in other variables. In fact, the correlation coefficient between intraindividual change in trait optimism and in state optimism was 0.21 for 1-week interval and 0.33 for 1-month interval in this study (see table S2), and 0.42 in Hoeppner et al. (2022). This seemingly increasing correlation appears in line with the abovementioned possibility, although further investigation is obviously needed.

In this study, state optimism measured by J-SOM showed significant intraindividual change in all intervals. To the best of our knowledge, no unusual events that would have a significant impact on Japan (e.g., a large earthquake) occurred during the period of the surveys (although there might have been effects of COVID-19; see below for discussion on this point). Thus, this study revealed for the first time that optimism measured as a state can show fluctuation within individual under daily life in the interval as short as few weeks, without any intervention. Such within-individual changes in state optimism tended to be larger than those of trait optimism in all intervals. This result demonstrates that, as predicted by the definition of the concept, state optimism is more susceptible to fluctuations influenced by events in daily life compared to trait optimism. The difference in fluctuations between state and trait optimism seemed to be smaller in the shortest (1 week) and relatively longer (4 weeks and 8 weeks) intervals, as compared to medium-length intervals (2 weeks and 3 weeks), based on the effect sizes. This result might also be in line with the abovementioned possibility that trait optimism reflects slow (long-term) changes in optimism (see Fig. S4-S5 and *Supplementary discussion* for the simulation results regarding this interpretation). Investigating this possibility directly is an important issue for future study. In addition, examining the detailed dynamics of fluctuations in state optimism and their mechanisms is considered another crucial future direction. For instance, if state optimism can be conceptualized as a modifiable prediction or belief about future, it is possible that its fluctuation is associated with differential impacts of positive and negative experiences (Palminteri & Lebreton, 2022; Sharot, 2011).

Measuring optimism as a state can help us determine the effectiveness of interventions aimed to increase optimism. In addition, we believe that measuring within-individual fluctuation of state optimism accurately and elucidating its nature is important to understand the relationship between optimism and various indices of mental health in more detail. Trait optimism has been known to be related to or predict a wide range of indices of mental health (Ames et al., 2015; Carbone & Echols, 2017; Miranda & Mennin, 2007; Rodrigues et al., 2021; Tanner et al., 2014; Vickers & Vogeltanz, 2000), but the specific mechanisms of such relationship remain largely unclear. Viewing optimism as time-varying state might help overcome this limitation by enabling to investigate temporally dynamic relationship between intraindividual change in optimism and in mental health. Especially, as stated in the introduction, we expect that treating state optimism as a future prospection that changes within individual through a learning process would contribute to elucidating the computational mechanisms of depressed mood and depression (Bennett et al., 2022; Eldar & Niv, 2015; Kube et al., 2020; Roepke & Seligman, 2016).

Several limitations of this study should be noted. One is that the quality of the daily life was measured by one non-validated item. We asked in this way because we considered it impossible to capture all personal events that might influence state optimism and aimed to measure the overall effect of the daily experience. Asking the occurrence of specific positive/negative events using scales (Clements & Turpin, 1996; Maybery et al., 2006) might help clarify what kind of events have strong influence on state optimism. The second point, somewhat related to the first point, is that we cannot completely rule out the possibility that some events might have caused population-level changes of state optimism during the survey period. Specifically, even though the impact on daily life had been decreasing, it cannot be ruled out that COVID-19 had some psychological effects on individuals during the data collection period (June to September 2022; see Fig. S3 and *Supplementary discussion* for population-level change in state optimism during the survey period and a detailed discussion on it). We might as well be cautious as to whether the range of fluctuations in optimism exhibited by the participants in this study was equivalent to the range that would be exhibited during normal times. The third point is that the sample was limited to university students. Given that the level of (trait) optimism might differ depending on age (Durbin et al., 2019; Kotter-Grühn & Smith, 2011), it is possible that fluctuation in state optimism has different characteristics depending on age and life stage. Generalizability of the present results should be examined by conducting a longitudinal survey on a wider range of populations. Finally, relatively small sample sizes in both surveys should be noted as another limitation, given the complex analyses used. We calculated the sample sizes assuming a simple correlation analysis, which might have been insufficient for small effect sizes in linear mixed models or Wilcoxon’s signed-rank tests. Results can be interpreted as preliminary, rather than definitive, and future studies attempting to replicate the present study should use more robust sample sizes.

Despite these limitations, this study confirmed the validity of newly developed J-SOM and found that state optimism can show change within individual in few weeks without any intervention. To the best of our knowledge, this is the first study to offer empirical evidence of intraindividual fluctuation in optimism under daily life. We believe focusing on state optimism will help determine the effectiveness of interventions aimed to increase optimism and clarify the specific mechanism underlying the relationship between optimism and various indices of physical/mental health.

### Supplementary Information

Below is the link to the electronic supplementary material.

Supplementary file1 (DOCX 1521 KB)

## Additional Information

### Availability of Data and Material

The data and codes that support the findings of this study are available at https://osf.io/rp5vh/. This study is not preregistered.
