# Supplementary material for: Intraindividual Fluctuation in Optimism Under Daily Life Circumstances: A Longitudinal Study
Source: Affect Sci. 2023 Nov 20;5(2):1–12. doi: 10.1007/s42761-023-00224-y (PMC11264638; doi:10.1007/s42761-023-00224-y)
Supplement: Supplementary file 1 — Supplementary file1 (DOCX 1521 KB) [file 42761_2023_224_MOESM1_ESM.docx]

**Supplementary information for “Intraindividual fluctuation in optimism under daily life circumstances: A longitudinal study”**

**Journal name**

*Affective Science*

**Authors & Affiliations**

Kanji Shimomura^1^*, Kenji Morita^1,2^, Yuki Nishiguchi^3^, Jeff C. Huffman^4,5^, Rachel A. Millstein^4,5^

1 Graduate School of Education, The University of Tokyo, Tokyo, Japan

2 International Research Center for Neurointelligence (WPI-IRCN), The University of Tokyo, Tokyo, Japan

3 Faculty of Education, Chiba University, Chiba, Japan

4 Department of Psychiatry, Massachusetts General Hospital, Boston MA, USA

5 Harvard Medical School, Boston MA, USA

*Corresponding author

Kanji Shimomura (skcpccp@gmail.com, ORCID: 0000-0003-4370-3710)

**Fig. S1**. Association between the intraindividual change in one month in J-SOM (state optimism) and in positive mood (a), in the quality of the previous month (b), in depressive mood (c), in anxiety mood (d), and in depressive symptoms (e). Magenta circles and cyan triangles represent the change in scores between “Wave 1 and Wave 2”, “Wave 3 and Wave 2”, respectively (the same applies to Figure S2).


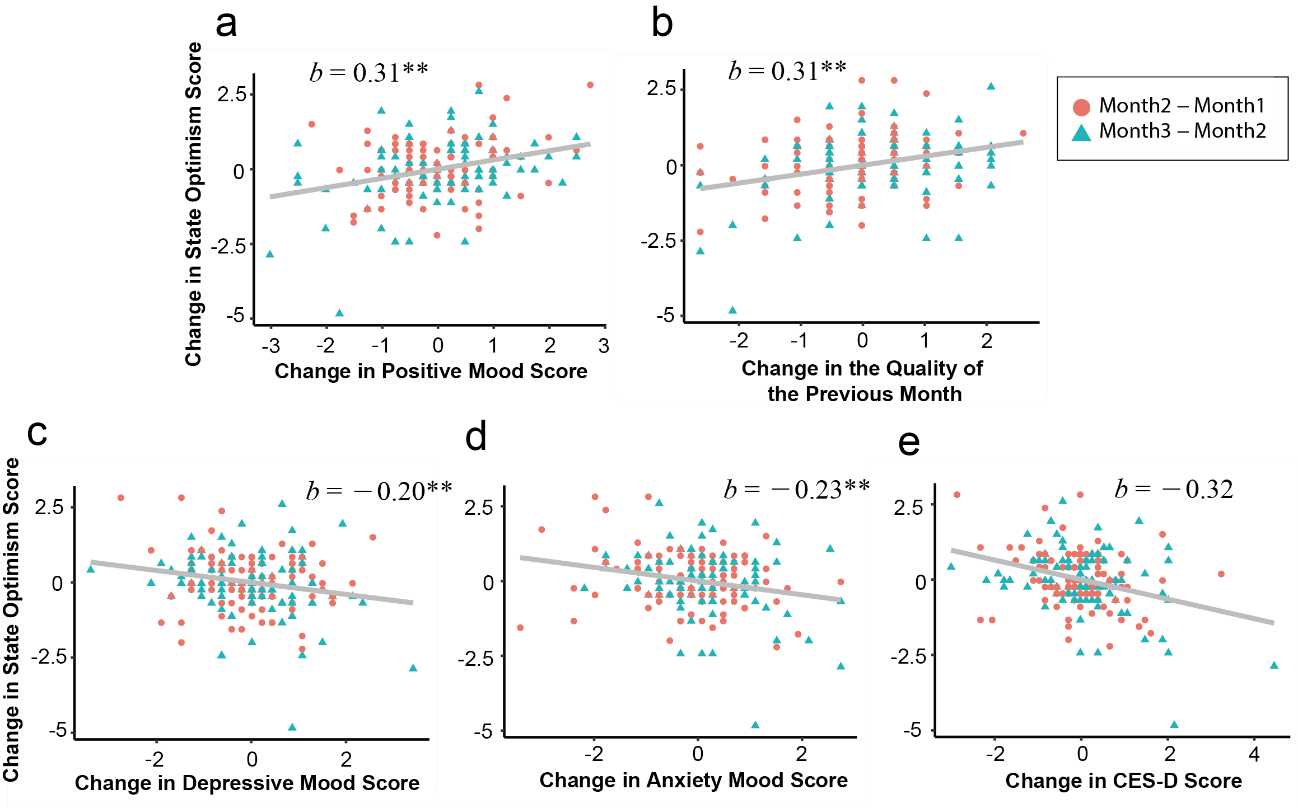


**Fig. S2**. Association between the intraindividual change in one month in LOT-R (trait optimism) and in positive mood (a), in the quality of the previous month (b), in depressive mood (c), in anxiety mood (d), and in depressive symptoms (e).


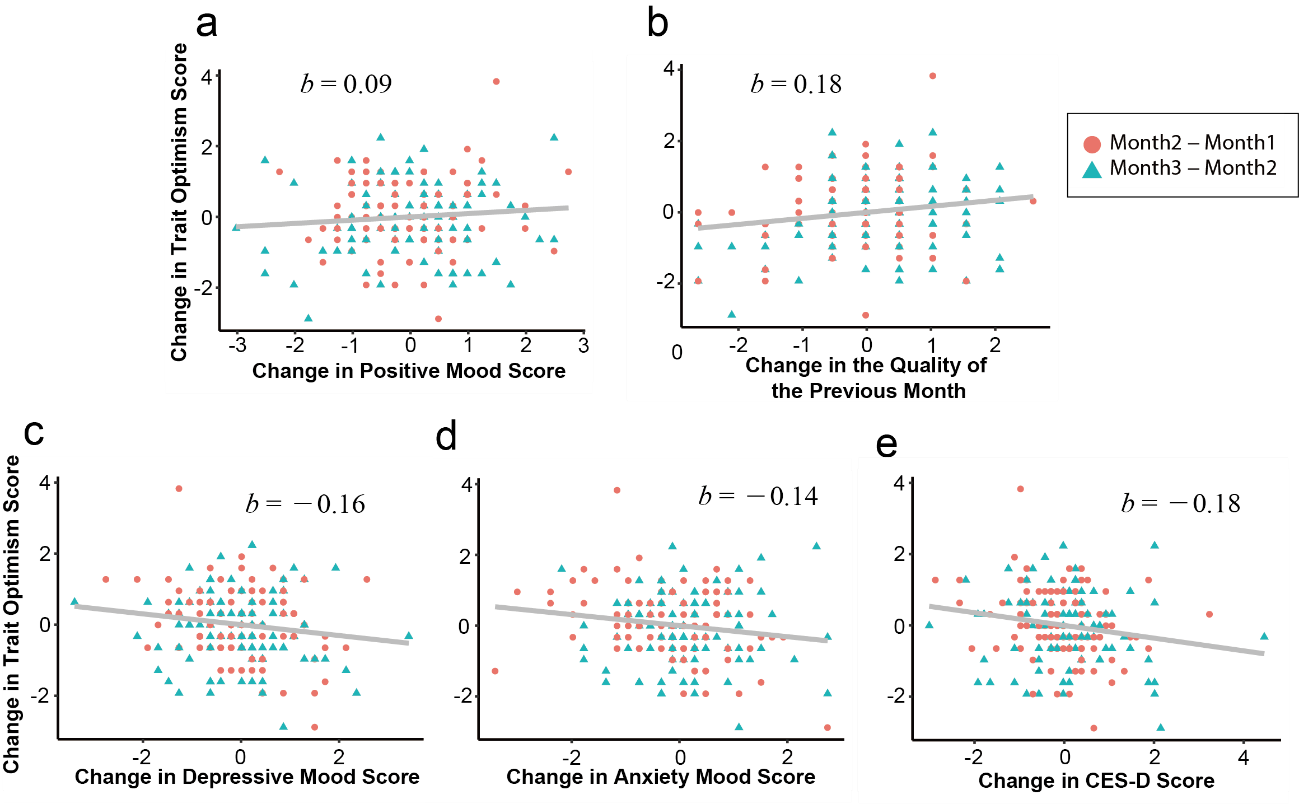


**Fig. S3**. Mean and SD of total score of J-SOM at each time point of 1w survey (a) and 1m survey (b). Asterisks on the horizontal line represent the p-value of multiple comparison (**p<.01).


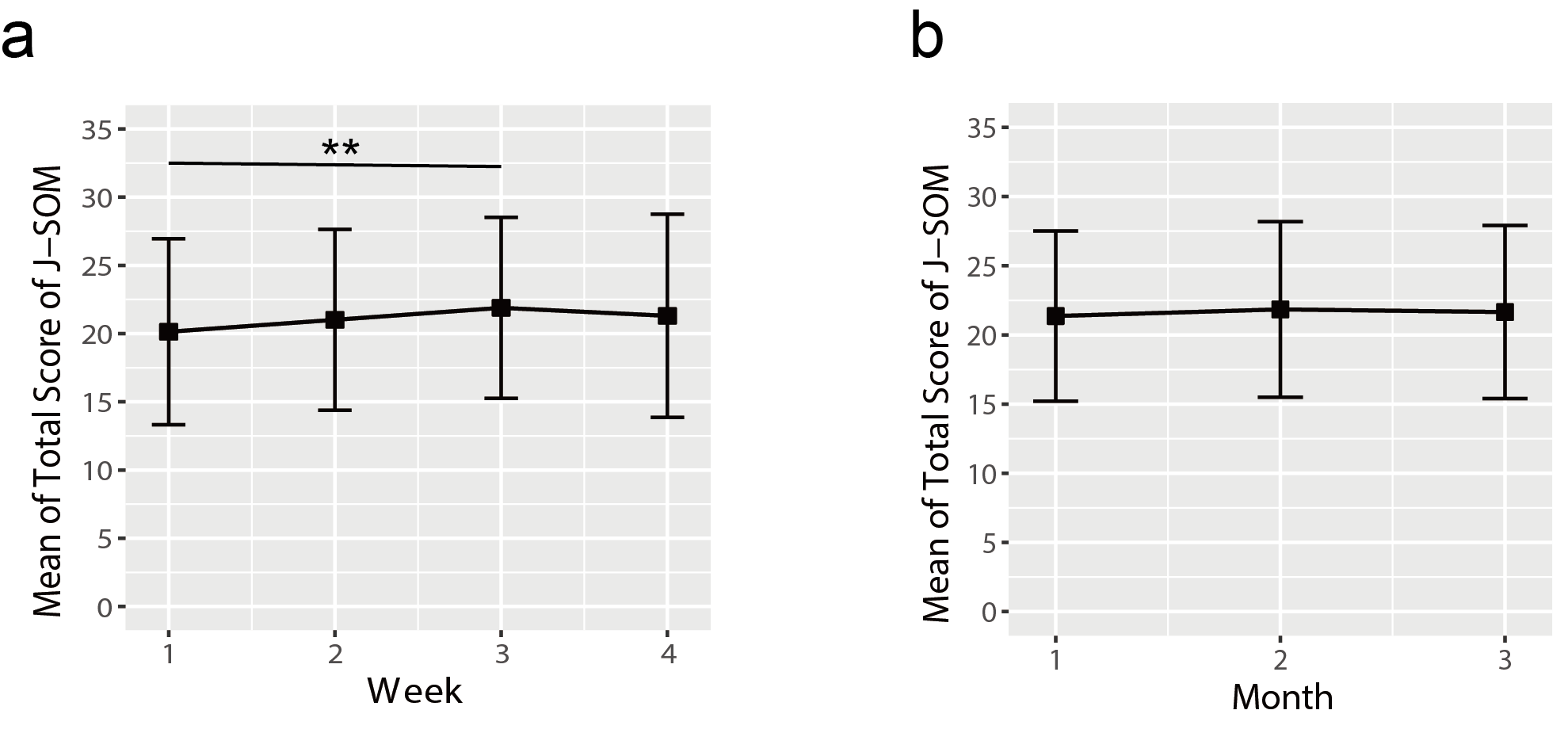


**Fig. S4**. Simulation results for the absolute changes in state/trait optimism and their difference. Red line represents state optimism and blue line represents trait optimism. (a) An example (i.e., a result in one simulation) change of state/trait optimism in 2000 days. A black dashed line represents Day 1001, which we used as the baseline day when we calculated the absolute change in trait/state optimism. (b) The mean absolute change in various intervals (calculated by “the value at current day – the value at the baseline day”) in state and trait optimism in 1000 days for 1000 simulations. Shading represents standard error (same applies to all), although it is hard to see in (b) and (d) because of the small size of SE. (c) The mean of the difference (state – trait) in the absolute change in various intervals in 1000 days. (d, e) The results for the first 100 days out of a total of 1000 days. Gray dashed lines in figure c and e represent the intervals used in the present study (i.e., 7, 14, 21, 28, and 56 days).


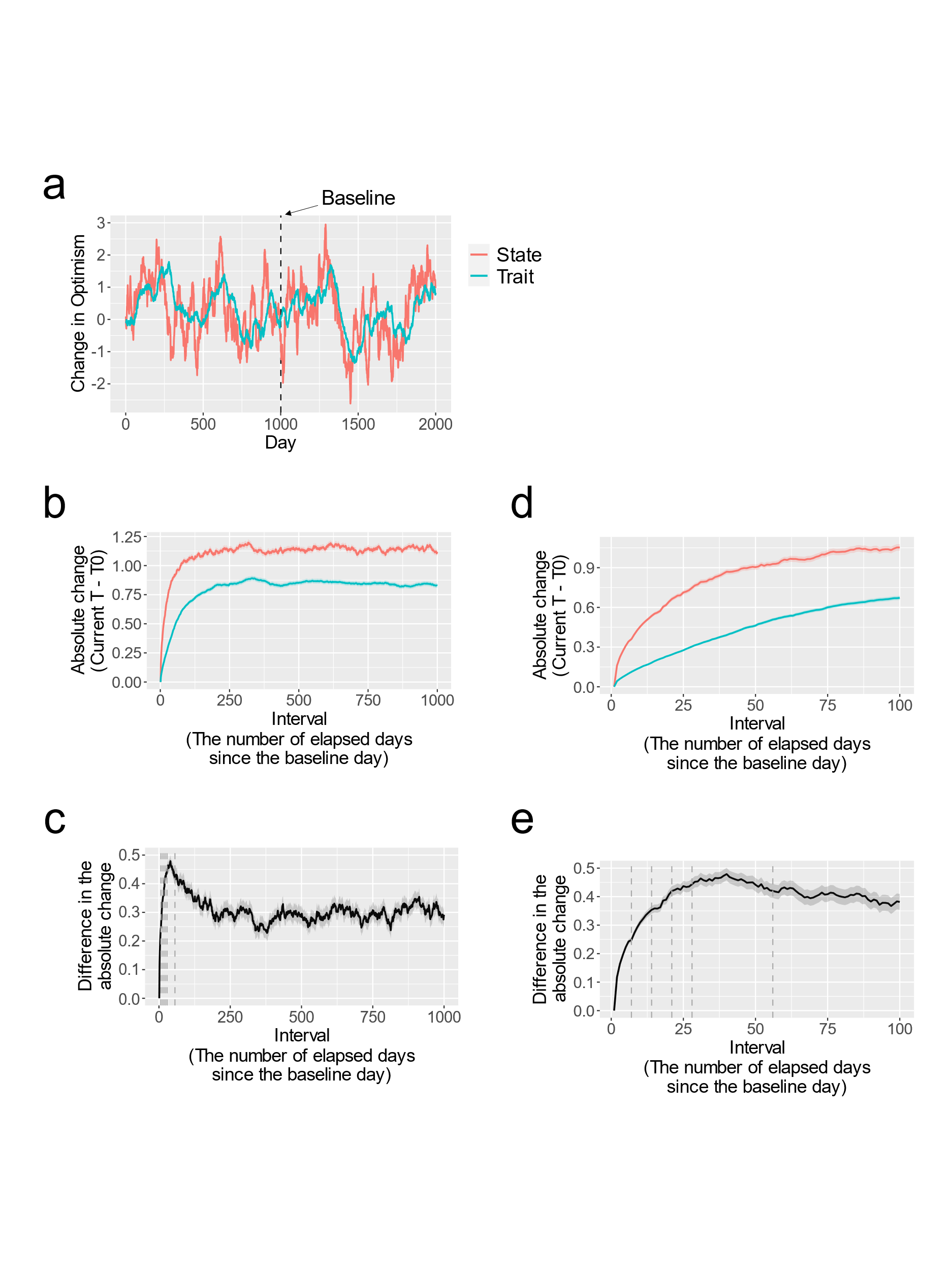


**Fig. S5**. Simulation results for the absolute changes in state/trait optimism and their difference with different length of moving average that was assumed in the calculation of trait optimism, which was set to 20 days in Fig.S4. Red line represents state optimism and blue line represents trait optimism. (a, b) Results for the length of 100 days. (c, d) Results for the length of 200 days. See the legend of Fig. S4 for the detailed explanation for the figures.


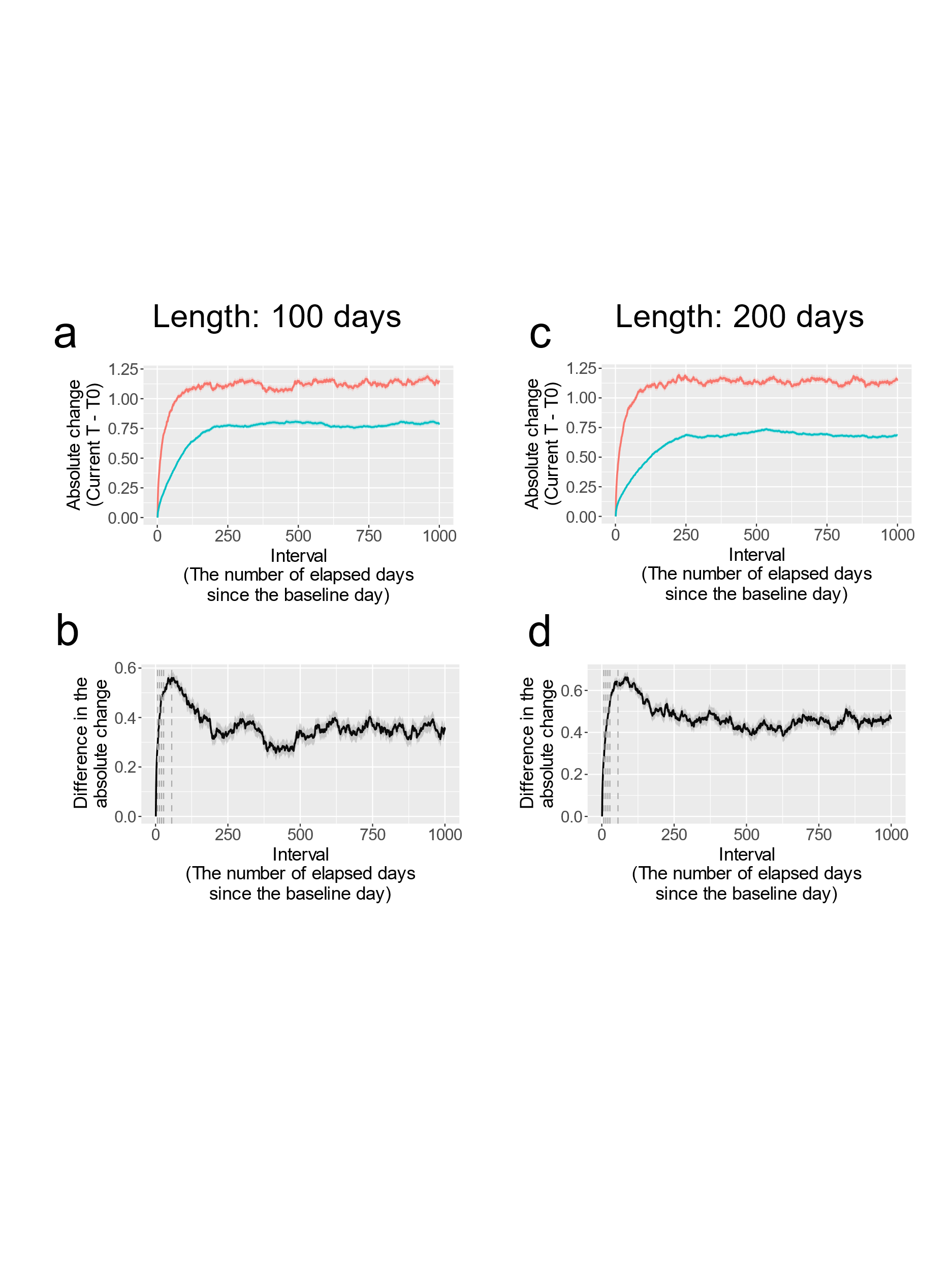


**Table S1**. Descriptive statistics of each scale and correlation matrix at wave 1 of 1m survey. J-SOM: Japanese version of State Optimism Measure, LOT-R: Life Orientation Test-Revised, SHS: Subjective Happiness Scale, DAMS-P: Positive Mood subscale DAMS, QM: The Quality of the previous Month, DAMS-D: Depressive Mood subscale of DAMS, DAMS-A: Anxiety Mood subscale of DAMS, CES-D: the Center for Epidemiological Studies Depression Scale

|  | Mean (SD) | Range | α | J-SOM | LOT-R | SHS | DAMS-P | QM | DAMS-D | DAMS-A | CES-D |
| --- | --- | --- | --- | --- | --- | --- | --- | --- | --- | --- | --- |
| State Optimism  (J-SOM) | 21.36 (6.15) | 7-35 | 0.88 | - | .72^***^ | .49^***^ | .52^***^ | .58^***^ | −.43^***^ | −.41^***^ | −.47^***^ |
| Trait Optimism (LOT-R) | 18.62 (4.46) | 6-30 | 0.72 |  | - | .46^***^ | .25^*^ | .33^**^ | −.39^***^ | −.39^***^ | −.37^***^ |
| Subjective Happiness (SHS) | 18.08 (2.83) | 4-28 | 0.78 |  |  | - | .43^***^ | .41^***^ | −.34^**^ | −.27^**^ | −.39^***^ |
| Positive Mood  (DAMS-P) | 13.63 (3.55) | 3-21 | 0.78 |  |  |  | - | .55^***^ | −.56^***^ | −.50^***^ | −.51^***^ |
| Quality of the previous month (QM) | 5.82 (1.66) | 1-9 | - |  |  |  |  | - | .45^***^ | −.33^**^ | −.48^***^ |
| Depressive Mood  (DAMS-D) | 10.90 (4.09) | 3-21 | 0.78 |  |  |  |  |  | - | .53^***^ | .79^***^ |
| Anxiety Mood  (DAMS-A) | 14.89 (4.04) | 3-21 | 0.85 |  |  |  |  |  |  | - | .48^***^ |
| Depressive Symptoms (CES-D) | 16.12 (9.65) | 0-60 | 0.87 |  |  |  |  |  |  |  | - |

*p<.05, **p<.01, ***p<.001

|  | one-week interval | |  | one-month interval | |
| --- | --- | --- | --- | --- | --- |
|  | J-SOM | LOT-R |  | J-SOM | LOT-R |
| LOT-R | 0.21*** |  |  | 0.33*** |  |
| Positive Mood (DAMS) | 0.45*** | −0.15* |  | 0.31*** | 0.09 |
| Quality of the previous week/month | 0.49*** | 0.08 |  | 0.30*** | 0.17* |
| Depressive Mood (DAMS) | −0.50*** | 0.05 |  | −0.20** | −0.15* |
| Anxiety Mood (DAMS) | −0.30*** | −0.13* |  | −0.23** | −0.15* |
| Depressive Symptoms (CES-D) | −0.45*** | −0.15* |  | −0.32*** | −0.17* |

**Table S2**. Raw correlations between the change in J-SOM/LOT-R and in other variables.

*p<.05, **p<.01, ***p<.001

**Table S3**. Median and mean±SD of the absolute change in mean scores of J-SOM and LOT-R in various intervals. Values of one-week, two-week, and three-week interval were obtained from the data of 1w survey, and values of four-week, eight-week interval were obtained from the data of 1m survey.

| Interval | J-SOM (state optimism) | LOT-R (trait optimism) |
| --- | --- | --- |
| 1 week | Med=0.29 (Mean=0.48±0.52) | Med=0.42 (Mean=0.33±0.27) |
| 2 weeks | Med=0.43 (Mean=0.62±0.57) | Med=0.33 (Mean=0.39±0.34) |
| 3 weeks | Med=0.43 (Mean=0.57±0.53) | Med=0.33 (Mean=0.38±0.33) |
| 4 weeks | Med=0.43 (Mean=0.47±0.39) | Med=0.33 (Mean=0.39±0.33) |
| 8 weeks | Med=0.43 (Mean=0.56±0.53) | Med=0.33 (Mean=0.43±0.33) |

**Supplementary discussion**

*Population-level change in state optimism*

In order to see if there was a population-level trend for the change in state optimism during the survey period, we used a mixed model with the total score of J-SOM as a dependent variable and each time point (i.e., wave) as an independent variable, assuming the random effect of individual on the intercept. Multiple comparison was conducted with “difflsmeans” function of R (lmerTest package). P-values of multiple comparison were manually adjusted using the Holm method.

Mean and SD of J-SOM at each time point of both surveys are shown in Figure S3. For 1w survey, each wave had marginally significant to significant positive effect on state optimism (week 2: *b*=0.96, *p*=.06, week 3: *b*=1.72, *p*<.001, week 4: *b*=1.29, *p*=.01). Multiple comparison revealed that there was a significant difference in “week 1 – week 3” score (*b*=$-$1.72, *p*<.01) and marginally significant difference in “week 1 – week 4” score (*b*=$-$1.29, *p*=.07), and no significant difference in any other pairs. These results indicate that the level of state optimism of the participants of 1w survey increased at the time of wave 3 and wave 4 compared with wave 1. For 1m survey, no wave had significant effect on state optimism (month 2: *b*=0.42, *p*=.40, month 3: *b*=0.30, *p*=.55). Multiple comparison also showed no significant difference in any of the pairs, indicating that there was no population-level change in state optimism during the period of 1m survey. We have two things to discuss based on these results.

The first one is about the possible effect of COVID-19. There was the seventh wave of coronavirus infection in Japan during July to August 2022. 1w survey and 1m survey were conducted during June 2022 and from the end of July to the beginning of September 2022, respectively. Therefore, if there was the effect of infection wave, it is expected that the level of state optimism would decline during 1m survey. But such decline was not observed. State optimism of the participants in 1w survey might also be expected to decline due to increasing anxiety towards the gradually rising number of infections, if there was the effect of infection wave. But rather than decreasing, it increased during 1w survey. One possible explanation for this is a diminished sense of crisis towards the pandemic. Unfortunately, though, we cannot determine whether this upward trend was caused by the effect of COVID-19 or by a possible impact of other events, because this study did not measure the external influence of specific events. It might also be an interesting future direction to investigate a potential naturalistic factor that leads to a population-level change in state optimism (e.g., effect of weather or season).

The second one is about the implication of the population-level increase in state optimism in 1w survey for the interpretation of the results shown in the main text. As stated in the main text, the absolute change in J-SOM was significantly larger than that of LOT-R in the interval of two (week 3 – week 1) and three weeks (week 4 – week 1). At the same time, there seems to be a population-level increase in J-SOM at week 3 and week 4 compared with week 1, according to the supplementary analysis. This raises the possibility that J-SOM shows larger intraindividual change than LOT-R only when there is a population-level change (i.e., only when there is an interindividual correlation in the direction of change). However, ad-hoc analysis revealed that the absolute change in J-SOM was larger than that of LOT-R also in the interval of “week 4 – week 2” (*Z*=2.60, *p*<.01, *r*=0.26) and of “week 3 – week 2” (*Z*=1.83, *p*=.03, *r*=0.19; note that only the results for the intervals based on “week 1” are shown in the main text). According to multiple comparison of the supplementary analysis, there was no population-level change in these intervals (Fig. S3). These results confirm that J-SOM shows larger intraindividual change than LOT-R even if there is no population-level tendency in the change.

*Examination on the validity of the assumption regarding the difference in the absolute change in state and trait optimism*

In our study, the difference in the absolute change in trait and state optimism seemed to be smaller in the shortest and relatively longer intervals, as compared to medium length intervals. In the Discussion, this result is interpreted as consistent with the possibility that trait optimism reflects long-term average of state optimism. This discussion is based on the assumption that “if trait optimism reflects long-term average of state optimism, then the difference in the absolute change in state and trait optimism is expected to decrease when the interval is long enough“. We tested whether this assumption is rational by conducting simulations in R (all code for conducting the simulation and drawing the figures are available at https://osf.io/rp5vh/).

We assumed that both state and trait optimism fluctuate at each time step (note that in this simulation, each time step corresponds to each day) in a Gaussian random walk with decay, which can be written in the following equation:

${Opt}_{t+1}=\lambda{Opt}_{t}+\left( 1-\lambda\right)\theta+\nu$

where ${Opt}_{t}$ represents the level of trait/state optimism on day *t*, and $\lambda$, $\theta$, and $\nu$ denotes decay rate, decay center, and diffusion noise, respectively. The decay center and diffusion noise were set differently for state and trait. Specifically, for state, the decay center was set to 0, while for trait, the decay center was set as a moving average of state optimism to reflect long-term change in state optimism. Additionally, while both state and trait had a zero-mean normal distribution for diffusion noise, the variance was set to be larger for state than for trait. We simulated the changes in state and trait optimism in 2000 days for 1000 times, and calculated the between-simulation mean, standard deviation, and standard error (SE) of the changes in trait/state optimism, the absolute change in various intervals with Day 1001 as reference, and the difference in the absolute change in various intervals. The initial values of state and trait optimism was randomly determined at each simulation using the same distribution as the diffusion noise of their own. We used Day 1001 (rather than Day 1) as reference because it is considered to be more natural to use initial values that are closer to the true state of the relationship between state and trait optimism, which is expected to be achieved by taking the values after certain time has passed, rather than to use the values generated from independent (normal) distributions. Fig. S4 shows the results of simulations for $\lambda$ set to 0.98, SD of the diffusion noise of state and trait optimism set to 0.2 and 0.05 respectively, and a length of moving average set to 20 days. As predicted, trait optimism showed smaller fluctuation than state optimism, in a way that tracks the long-term change in state optimism (Fig. S4a). Notably, the absolute change in both state and trait optimism increased up to a certain length of interval (about 250 days) and then they both became relatively stable beyond that point, indicating that they reached their asymptotic value (Fig. S4b). And as a result, the difference in the absolute change increased during the initial shorter intervals but started to decrease as the interval exceeded around 30 days, eventually being stable in the intervals longer than approximately 250 days (Fig. S4c). When focusing on the intervals up to 100 days only, which is closer to those targeted in this research, the absolute changes in state optimism exhibited a rapid increase in shorter intervals, followed by a gradual increase. In contrast, the absolute changes in trait optimism continued to increase gradually during these intervals (Fig. S4d). Consequently, as mentioned earlier, the difference between them increased for intervals of up to about 30 days but then gradually decreased (Fig. S4e). The features of these results were preserved when the length of moving average was set longer (Fig. S5), although the interval at which the difference begins to decrease and to become stable slightly differ depending on the length.

In summary, under the assumption that trait optimism reflects the long-term average of state optimism, the difference in the absolute change (1) increased for the shortest intervals, and (2) then decreased for short to medium intervals, and (3) eventually became relatively constant beyond a certain length of the interval. Specifically, with the parameter sets in the simulation of Fig. S4, the difference increased for the intervals of one week to four weeks, while it decreased in the interval of eight weeks. Although not all simulation results match the actual ones, these results confirmed that when the interval gets longer, the difference in the absolute difference can get smaller as compared to when the interval is short. It is an interesting future direction to test whether we can observe the patterns expected from these simulations by conducting longitudinal surveys over a longer period than the present study.
